# Standardization of Therapeutic Measures in Antibiotic Consumption Monitoring to Compare Different Livestock Populations

**DOI:** 10.3389/fvets.2020.00425

**Published:** 2020-07-23

**Authors:** Katharina Hommerich, Charlotte Vogel, Svetlana Kasabova, Maria Hartmann, Lothar Kreienbrock

**Affiliations:** Department of Biometry, Epidemiology and Information Processing, WHO Collaborating Centre for Research and Training for Health at the Human-Animal-Environment Interface, University for Veterinary Medicine Hannover, Hanover, Germany

**Keywords:** antimicrobial usage, livestock, confounding, stratification, animal demographics, population correction unit

## Abstract

Using sales data, information on antimicrobial consumption in animals is collected cumulatively across the European Union and member countries of the European Economic Area, which is documented and reported by every country and published within annual reports by the European Surveillance of Veterinary Antimicrobial Consumption (ESVAC). These serve to perform cross-border comparisons of antimicrobial consumption, despite their ambiguity due to the different units and key figures used. To improve comparability, the European Medicines Agency has introduced the population correction unit (PCU), which represents the biomass of a livestock population and is related to antibiotic consumption. However, the PCU does not consider the variability of how a livestock population is composed structurally regarding the proportions of production types contained therein. To achieve better comparability between the different geographical areas, we therefore applied a system of standardization in different examples and in real antimicrobial consumption data. This was done by quantifying the consumption of antibiotics by livestock in exemplary regions and countries (Denmark, Germany, France) by means of the active substance used (mg/kg) and subjecting it to a direct and indirect standardization procedure to identify and measure differences in consumption in relation to the composition of livestock demographics. The consideration of livestock demographics results in substantial effects when comparing antimicrobial usage in livestock. To achieve a more compelling comparability in the context of monitoring antibiotic consumption in livestock populations, we recommend using an indirect standardization method, to control potential confounding effects caused by different livestock demographics. This assumes that animal populations can be structured accordingly well. Correspondingly, detailed information on antimicrobial usage by species should be available for this type of stratification.

## Introduction

The monitoring and surveillance of antimicrobial usage (AMU) is essential for identifying factors that drive the development of antimicrobial resistance in humans and animals, one of the major issues defined by the World Health Organization that threatens global health (1). For Europe, there is no binding legislation with regard to the implementation of monitoring programs at the national level. Different countries use their own specifications with regard to collection and evaluation of AMU data at the farm level and the definition of standard weights and populations (2). With respect to the implementation of synchronized monitoring programs at the national level, countries are still at different levels (3). In a comparison of AMU data from European countries and the United States of America, large differences have been found, which were attributed to the availability of data and differences in livestock demographics (4). Other stakeholders have also drawn attention to the need for the harmonization and standardization of AMU data at the farm level. For instance, the AACTING network has issued guidelines to provide useful support when designing or revising farm-level AMU monitoring systems (5). However, at this point, if data are available, they are usually not standardized internationally and are therefore not unambiguously comparable due to the differences in calculation methods and units (6).

Therefore, to perform cross-border comparisons of AMU, sales data are typically used, documented and reported by every country (7). Information on which antimicrobial agents are sold across the European Union (EU) are collected, evaluated and published in annual reports (8). The ESVAC project (European Surveillance of Veterinary Antimicrobial Consumption) was launched by the European Medicines Agency (EMA), following a request from the European Commission, to develop a harmonized approach for the collection of data on the AMU in animals from the EU and the European Economic Area Member States (9). The quantities of active substances are expressed in mg/PCU (population correction unit, a representation of the biomass of all farm animals within an entire national livestock), and thus, a comparison between the countries is possible (9). Suggestions have already been made to adjust the PCU by reevaluating the standard animal weight and including farm animal lifespan (10). Bondt et al. compared the overall exposures of the animals using model calculations and the assumption of varying treatment incidences in two countries. This comparative analysis of sales figures from Denmark and the Netherlands showed that reliable results can only be obtained based on consumption per species and that it is therefore necessary to have information on the animal population separated by species (11). Nevertheless, the antibiotic use and consumed amounts of almost all active substance groups differ between countries, which can also be attributed to the fact that the proportions of the various animal species differ between countries. As the types and incidences of infectious diseases vary considerably between animal species and production category (e.g., beef vs. dairy cattle), consequently, the sales of veterinary antibacterial agents are thought to be influenced by animal species demographics (7). Because livestock populations of the different countries are composed differently, comparisons may be biased to higher consumption for countries that maintain more treatment-intensive production types of livestock. As an example, countries with a high proportion of fattening pigs had a higher consumption per PCU (12). Although sales data are available in most European countries, there is a lack of AMU surveillance at the animal species level in many countries. Therefore, at present, population-based evaluations are carried out with regard to their distribution of the PCU by animal species and country (13), but these are not linked to information on species-related antimicrobial consumption.

To illustrate the effect of the population composition and the corresponding variation in the use of the active ingredients in international comparisons of AMU, we applied standardization techniques to several data sets, i.e., to artificial data to illustrate the strategy, to real antibiotic consumption data, which were documented within the VetCAb-S study (Veterinary Consumption of Antibiotics—Sentinel), a German antibiotic monitoring sentinel, and to international data from some European countries.

## Materials and Methods

Compelling comparisons of AMU between two different populations can only be made if the populations are similar with respect to characteristics that might affect AMU. If the populations are dissimilar with respect to such characteristics, erroneous conclusions might be drawn because these characteristics may act as confounders (14). These confounders can be prevented by standardization (15). Standardization is a method used to compare observed and expected rates of a given outcome by removing the influence of factors that may confound the comparison (16). To achieve a better comparability between the consumption of antibiotics in different geographical areas regarding their different populations and corresponding exposure (17), we applied the systematics of direct and indirect standardization in example data and subsequently transferred these to real AMU data from a German antibiotic monitoring sentinel (VetCAb-S) as well as to AMU-data derived from national reports from Denmark (18) and France (19).

### Basic Example - Which Data Are Required?

Consider two hypothetical regions A and B with different livestock demographics. Table 1 shows the characteristics of the standard population in Region A (hereafter marked with an asterisk “^*^”) and of the study population in Region B. For the selection of the standard population, sufficient information on the characteristic to be examined should be known. The choice of the reference population should be realistic and relevant with regard to the planned evaluations (14), e.g., that the region is considered reasonably representative at regional or national level. The populations to be compared were divided into animal strata (15). The term “strata” defines the livestock demographics broken down into different layers of production types of livestock animals. The expression “production type” hereafter describes the type of use of the livestock animal within an animal species, for example: dairy cows kept for milk production or beef cattle reared for meat production. In the example considered, livestock within one region is composed of K strata, where here K = 2, consisting in this case of pigs (k1) and cattle (k2). The different strata each make up a proportion of w*_k_ (k = 1 … K) in the total population. By definition, *w*_k_* denotes the proportion of the *k*-th production type in Region A (standard population) and *w*_*k*_ in Region B (population under study). Note that proportions add up to 1, i.e., ∑*w*_k_* = 1. Suppose that pigs are usually treated with 80 mg/kg (*AMU*_1_*) active substance in Region A, whereas cattle are treated with 20 mg/kg (*AMU*_2_*). This antimicrobial consumption information *AMU*_*k*_ is also required for the study population, with which the comparison will be performed. For each stratum, weighted AMU-amounts can be calculated by multiplying the proportion by its production-type specific quantity of antimicrobials used. Then, the overall amount of active treatment equals the sum over all weighted AMU-amounts from the total population (see Table 1).

(1) $$\begin{array}{r} AMU^{*}=\sum w_{k}^{\;*}\cdot AMU_{k}^{\;*} \end{array}$$

Similarly, in the study population, the overall amount is determined as

(2) $$\begin{array}{r} AMU=\sum w_{k}\cdot AMU_{k} \end{array}$$

This forms the basis for the calculation of the “Treatment Ratio” (TR), which compares the individual overall amount by forming a ratio as

(3) $$\begin{array}{r} Treatment\;Ratio=\;\frac{AMU}{AMU^{*}} \end{array}$$

Here, the Treatment Ratio = 42/62 = 0.68; the overall amount is lower in the study population in Region B than in the standard population in Region A. Thus, without considering the livestock demographics as a confounding factor, Region B consumes 0.68 of the antibiotic active ingredients consumed in Region A (i.e., Region B consumes 32% less than Region A).

**Table 1:** Livestock demographics and treatment in Region A and Region B.

**REGION A (STANDARD POPULATION)**				
**Production type**	**k**	***w***_k_*	***AMU***_k_* **in mg/kg**	Weighted AMU*
Pigs	1	*w*_1_* = 0.70	80	56
Cattle	2	*w*_2_* = 0.30	20	6
				AMU* = 62
**REGION B (STUDY POPULATION)**				
**Production type**	**k**	***w***_***k***_	***AMU***_***k***_ **in mg/kg**	**Weighted AMU**
Pigs	1	*w*_1_ = 0.40	60	24
Cattle	2	*w*_2_ = 0.60	30	18
				*AMU* = 42

### Implementation of Standardization Procedure

For direct standardization, the observed proportions in the individual strata of the standard population are assumed to be fixed as the true underlying distribution of the production types to identify population-structural differences and their impact on antibiotic consumption. In Table 2, the region-specific proportions of the production types of the standard population within Region A are applied to the study population within Region B. The transferred population stratification is weighted with the applied amount of active ingredients in mg/kg of the study population (Region B). The sum of these values is

(4) $$\begin{array}{r} AMU_{st}=\sum w_{k}^{\;*}\cdot AMU_{k} \end{array}$$

which yields the total amount of active ingredients that would be estimated if the study population had the livestock demographics as our standard population. This is therefore denoted as *AMU*_*st*_ for the standardized AMU-amount.

**Table 2:** Direct standardization of Region B.

**Production type**	**k**	w_k_*	**AMU_k_ in mg/kg (Region B)**	**Weighted, standardized AMU**
Pigs	1	*w*_1_* = 0.70	60	42
Cattle	2	*w*_2_* = 0.30	30	9
				*AMU*_*st*_ = 51

Following (4), the “Comparative Treatment Figure” (CTF) is determined by comparing the directly standardized AMU of the study population with the total AMU of the standard population.

(5) $$\begin{array}{r} CTF=\frac{AMU_{st}}{AMU^{*}}=\frac{\sum w_{k}^{\;*}\cdot AMU_{k}}{\sum w_{k}^{\;*}\cdot AMU_{k}^{\;*}} \end{array}$$

This measure indicates the ratio in the population stratification when assuming the same structural proportions as in the study population.

Here, the Comparative Treatment Figure = 51/62 = 0.82; considering the population stratification as in the standard population, Region B consumes 0.82 of the antibiotic active ingredients consumed in Region A (i.e., Region B consumes 18% less than Region A).

Similarly, the procedure can be performed using an indirect standardization technique. This technique appears more suitable here, as it is easier to assume similar treatment regimens in different regions. In this regard, we weight the region-specific proportions of the production types of the standard population (Region B) with the applied amount of active ingredients in mg/kg of the study population (Region A) (see Table 3).

(6) $$\begin{array}{r} AMU_{expected}=\sum w_{k}\cdot AMU_{k}^{\;*} \end{array}$$

By now comparing the nonstandardized AMU with the AMU_expected_, the “Standardized Treatment Ratio” (STR) is obtained by

(7) $$\begin{array}{r} STR=\frac{AMU}{AMU_{expected}}=\frac{\sum w_{k}\cdot AMU_{k}}{\sum w_{k}\cdot AMU_{k}^{\;*}} \end{array}$$

In the present example, this precisely means that the AMU rate of the study population is related to the expected AMU rate. This indicates the rate in overall therapy when assuming the same treatment regime as in the standard population.

**Table 3:** Indirect standardization of Region B.

**Production type**	**k**	**w_k_**	AMU^*^ in mg/kg (Region A)	**Weighted, expected AMU**
Pigs	1	*w*_1_ = 0.40	80	32
Cattle	2	*w*_2_ = 0.60	20	12
				*AMU*_*expected*_ = 44

Here, the Standardized Treatment Ratio = 42/44 = 0.95; assuming the same treatment regimen in Region B as in Region A, Region B consumes 0.95 of the antibiotic active ingredients consumed in Region A (i.e., Region B consumes 5% less than Region A).

### Transferring the Method to VetCAb-S Data

To quantify antibiotic consumption, real AMU data collected within the scientific project VetCAb-S were used. The aim of the study is to evaluate and describe the use of antibiotics in farm animals in Germany and to assess it on a scientific basis (20, 21). Since 2013, the project has continued as a longitudinal study with ongoing participant recruitment and data collection (22, 23). Participating veterinarians and farmers voluntarily provide information on AMU at the farm level by official application and delivery forms, which are transferred into a database that maintains information about the species, production type, number of animals treated, the treatment date and duration and the name and amount of the medicinal product used.

For this exercise, subsets from real antibiotic consumption data from the VetCAb-S study were formed. To ensure a cross-sectional study-like study population, the data were checked for representativeness by investigating the demographic characteristics of the participating farms by comparing this data with official data from agricultural statistics (24).

Here, we systematically selected areas with a high density of pig farms and a low density of cattle farms and vice versa from the VetCAb-S population. For this purpose, the county codes of all VetCAb regions, defined by Merle et al. (25), were analyzed with regard to the number of participating cattle and pig farms and their stable capacities. To determine the total biomass of livestock kept in the defined subregions within the considered time period (half a year), the numbers of livestock animals kept on the considered farms were multiplied by the estimated weight at treatment. Therefore, the standard weights defined by ESVAC were used; i.e., for fattening pigs 65 kg, for calves 140 kg, and for beef cattle and dairy cows 425 kg (9). In addition, in order to adjust the determined biomass of fattening pigs, it was multiplied by the usual passage rate for Germany for half a year of 1.425 (26). Based on the determined biomass, the corresponding proportions (k) of the total livestock were determined. The quantities of antimicrobial active substances consumed were determined for the corresponding period and subregions to be compared. The consumption of antibiotics by livestock in the exemplary subregions was measured by the ratio of the biomass in kg and the amount of active ingredients used in mg. After addition of the respective average AMU of the individual production types, the overall AMU of the active ingredient for the entire livestock population was obtained (see Table 4) to then determine the treatment ratio of the two regions.

**Table 4:** Livestock demographics and treatment, VetCAb-S Subregion 1 and Subregion 2.

**SUBREGION 1**					
**Production type**	**Biomass (kg)**	${\text{w}}_{\text{k}}^{*}$	**Active ingredients (mg)**	${\text{A}M\text{U}}_{\text{k}}^{*}$ **in mg/kg**	Weighted AMU*
Fattening pigs	17,685,910	76.9	1,416,595,566	80.1	61.6
Dairy cows	3,224,900	14.0	32,157,300	10.0	01.4
Calves	946,820	04.1	26,669,911	28.2	01.2
Beef cattle	1,150,900	05.0	13,126,236	11.4	00.6
∑	23,008,530	100.0	1,488,549,013		64.7
**SUBREGION 2**					
**Production type**	**Biomass (kg)**	**w**_**k**_	**Active ingredients (mg)**	**AMU**_**k**_ **in mg/kg**	**Weighted AMU**
Fattening pigs	5,142,633	52.4	294,047,708	57.2	30.0
Dairy cows	3,077,425	31.3	24,432,104	07.9	02.5
Calves	1,011,140	10.3	34,603,400	34.2	03.5
Beef cattle	586,500	06.0	1,213,250	02.1	00.1
∑	9,817,698	100.0	354,296,462		36.1

### VetCAb Study Population

In accordance with the example above, the direct and indirect standardization method was applied (see Tables 5, 6), and the “Comparative Treatment Figure” and “Standardized Treatment Ratio” were determined according to Equations (5) and (7). For the analyses, data from the second half of the year 2014 were selected to demonstrate the method with real-application data. The two selected subregions are located in the middle and northwest of Germany, both of which employ intensive pig farming. Within these subregions, one was identified with a small and one with a large proportion of cattle. Subregion 1 is made up of 79 dairy farms, 41 beef cattle farms and 179 pig farms with 17,059 livestock places for dairy cows, calves and beef cattle (hereafter summarized as “cattle”) and 190,941 livestock places for fattening pigs. Subregion 2 comprises 69 dairy farms, 25 beef cattle farms and 52 pig farms, with 15,772 livestock places for cattle and 55,521 for fattening pigs. Biomass (in kg) is defined here as the inventory of livestock that was kept at the farms within the defined regions during the study period.

**Table 5:** Direct standardization of VetCAb-S Subregion 2.

**Production type**	${\text{w}}_{\text{k}}^{*}\;$ Subregion 1	**AMU_k_ in mg/kg Subregion 2**	**Weighted, standardized AMU**
Fattening pigs	76.9	57.2	44.0
Dairy cows	14.0	07.9	01.1
Calves	04.1	34.2	01.4
Beef cattle	05.0	02.1	00.1
∑	100.0		46.6

**Table 6:** Indirect standardization of VetCAb-S Subregion 2.

**Production type**	**w_k_ Subregion 2**	${\text{A}M\text{U}}_{\text{k}}^{*}$ in mg/kg Subregion 1	**Weighted, expected AMU**
Fattening pigs	52.4	80.1	42.0
Dairy cows	31.3	10.0	03.1
Calves	10.3	28.2	02.9
Beef cattle	06.0	11.4	00.7
∑	100.0		48.7

The biomass of Subregion 1 is accordingly divided into 77% pigs and 23% cattle and is hereafter referred to as the “pig dense region.” The biomass of Subregion 2 is distributed into approximately equal parts with 52% cattle and 48% pigs, hereafter referred to as the “species balanced region.” The respective overall amount of active substance used per production type and subregion are shown in Table 4.

### Transferring the Method to EU AMU Data

As a last exercise the standardization technique presented is applied to European antibiotic consumption data. For this purpose, it was assumed that livestock demographics are composed of pigs and cattle only and their percentages shown in Table 7 were extrapolated, to 100% therefore (see Table 8) (27). It should be noted that after extrapolation to 100%, the stratification of the livestock population no longer corresponds directly to the real country-specific French, German or Danish livestock population. Data on antibiotic consumption for Germany were obtained from the VetCAb collective, Subregion 1 (see Table 4). Due to the clear differences in the percentage distribution of the species cattle and pig within Table 7, AMU-data data suitable for the application of the methodology for France and Denmark were obtained from reports published annually (18, 19). Germany artificially serves as the trial standard for the calculations; France and Denmark form the study populations.

**Table 7:** Livestock in tons and percentages of total population in BE, DK, DE, ES, FR, NL, and the UK in 2014 (27).

**Country**	**Cattle**		**Pigs**		**Poultry**		**Sheep**		**Total livestock (tons)**
	**tons**	**%**	**tons**	**%**	**tons**	**%**	**tons**	**%**	
Belgium	1,052,725	71.4	412,750	28.0	8,442	0.6			1,473,917
Denmark	660,025	44.3	826,085	55.5	3,260	0.2			1,489,370
Germany	5,415,350	72.9	1,842,035	24.8	50,849	0.7	120,075	1.6	7,428,309
Spain	2,583,575	46.9	1,726,920	31.4	39,182	0.7	1,157,400	21.0	5,507,077
France	8,190,175	85.0	864,500	9.0	47,306	0.5	537,600	5.6	9,639,581
Netherlands	1,771,825	66.4	784,225	29.4	31,356	1.2	80,250	3.0	2,667,656
United Kingdom	4,119,525	67.0	293,150	4.8	37,853	0.6	1,701,525	27.7	6,152,053

*The following estimated weights at treatment by ESVAC were used: Cattle 425 kg, Pig 65 kg, Poultry 1 kg, Sheep 75 kg (7)*.

**Table 8:** Livestock demographics (27) and AMU-data, German Subregion 1 (VetCAb-S), France (19) and Denmark (18).

**Production type**	${\text{w}}_{\text{k}}^{*}$	${\text{A}M\text{U}}_{\text{k}}^{*}$ in mg/kg	Weighted AMU^*^
**Germany (Standard population)**			
Pigs	0.25	80.1	20.0
Cattle	0.75	13.5	10.1
			AMU^*^ = 30.2
**France (Study population)**			
	**w**_**k**_	**AMU**_**k**_ **in mg/kg**	**Weighted AMU**
Pigs	0.10	64.3	6.4
Cattle	0.90	14.1	12.7
			*AMU* = 19.1
**Denmark (Study population)**			
Pigs	0.56	91.0^*^	50.9
Cattle	0.44	19.0^*^	8.4
			*AMU* = 59.3

* *source of data: biomass (27), kg active compound (18)*.

## Results

### Standardization of VetCAb Data

After multiplying the amount of active substances per production type in each subregion by the percentage distribution of production types, the weighted amount of active substances was 64.7 mg/kg in Subregion 1 and 36.1 mg/kg in Subregion 2, with a resulting Treatment Ratio of 0.56 (see Table 4). This quotient means that in Subregion 2, in relation to Subregion 1, slightly more than half as much active substance is consumed (i.e., Subregion 2 consumes 44% less than Subregion 1).

Following direct standardization, the biomass distribution in Subregion 2 is set the same as in Subregion 1 (see Table 5). Consequently, the resulting Comparative Treatment Figure was 0.72; i.e., considering the same population stratification as in the standard population, Subregion 2 consumes 72% of the antibiotic active ingredients consumed in Subregion 1 (in other words Subregion 2 consumes 28% less than Subregion 1).

If the concept of indirect standardization is used for Subregion 2, the treatment habits of Subregion 1 are assumed. This yield expected overall AMU outlined in Table 6. Subsequently, the resulting Standardized Treatment Ratio was 0.74, which means that assuming the same AMU in species balanced Subregion 2 as in the standard population in Subregion 1; the total amount of active substance consumed is 0.26% lower in Subregion 2.

### Standardization of EU AMU Data

The comparison between Germany (standard population) and Denmark (study population) results in a TR of 59.3/30.2 = 2.0 i.e., in the example Denmark consumes 2 times the antibiotic active ingredient consumed in Germany. After applying the direct standardization method, the CTF = 37.0/30.2 = 1.2. Considering the population stratification as in Germany, Denmark consumes 120% of the antibiotic active ingredients consumed in Germany. After applying the indirect standardization method, the STR = 59.3/50.8 = 1.2. Assuming the same treatment regimen in Denmark as in Germany, Denmark consumes 120% of the antibiotic active ingredients consumed in Germany.

The comparison between Germany (standard population) and France (study population) results in a TR of 19.1/30.2 = 0.6 (i.e., France consumes 40% less than Germany). After applying the direct standardization method, the CTF = 26.6/30.2 = 0.9 “(i.e., France consumes 10% less than Germany). After applying the indirect standardization method, the STR = 19.1/20.2 = 0.9 (i.e., France consumes 10% less than Germany) (see Tables 8–10 respectively).

**Table 9:** Direct standardization of AMU-data, France and Denmark.

**Production type**	${\text{w}}_{\text{k}}^{*}$	**AMU_k_ in mg/kg**	**Weighted, standardized AMU**
**France**			
Pigs	0.25	64.3	16.1
Cattle	0.75	14.1	10.6
Total			*AMU*_*st*_ = 26.6
**Denmark**			
Pigs	0.25	91.0	22.7
Cattle	0.75	19.0	14.3
			*AMU*_*st*_ = 37.0

**Table 10:** Indirect standardization of AMU-data, France and Denmark.

**Production type**	**w_k_**	AMU^*^ in mg/kg	**Weighted, expected AMU**
**France**			
Pigs	0.10	80.1	8.0
Cattle	0.90	13.5	12.2
			*AMU*_*expected*_ = 20.2
**Denmark**			
Pigs	0.56	80.1	44.9
Cattle	0.44	13.5	5.9
			*AMU*_*expected*_ = 50.8

## Discussion

To assess the risk of the development of antimicrobial resistance, a precise quantification of AMU is indispensable. It is therefore necessary to generate access to detailed information on antimicrobial usage. Generally, to report the antimicrobial consumption of a country, national requirements, such as the quantification of use data, as well as international documentation and comparison of quantities of antimicrobial active ingredients sold must be followed (28). According to these, the overall amount of the active ingredient sold in target animal populations is recorded cumulatively and published in annual reports in the context of the ESVAC project by the European Medicines Agency (7). To quantify livestock, in the monitoring of AMU, the biomass or another equivalent, such as the PCU, is usually calculated (7). The PCU figure is a harmonized average weight in kilograms of all animals at the time of treatment multiplied by the number of animals based on national statistics (7). Regardless of the species in question, the weight in kilograms of the livestock is consequently considered as equal.

Since data collection by production strata is recommended by the EMA (29) but has not yet been implemented, the classical standardization procedure is to adjust for the confounding of a stratification variable. This established method has thus served in many fields of standardization within human populations (15). Using this standardization technique, different rates are determined to allow more in-depth comparisons of the antibiotic consumption of a population based on its composition. The generated key figures are artificial measures that cannot be interpreted on their own but only make sense in comparison with a second rate (15).

By using the direct standardization method, a standard distribution of the population in each stratum of the confounder for the factor of interest is needed. This approach is very popular in human medicine and demography, e.g., for the standardization of mortality rates. By nature of the method, the standard population is arbitrary, but usually “average populations” are used to calculate standard rates, which are close to the real world. A well-established standard therefore is Segi's world population (30), but other standards like European or African standard populations are used as well. Here, the selection of the populations to be compared made is intended only to illustrate the methodology.

Applying the indirect standardization method and computing standardized ratios (STR) is a more often used method to control potential confounding effects when comparing rates from different populations (15). These are based on a set of stratum-specific rates from the standard population (here the species-specific rates) together with the observed proportion of the treatment behavior in each of the strata of the study population. This method is especially useful if the actual stratum-specific rates (in this case the species-breakdown) are not available for the study population (15). The indirect standardization method could therefore be used to predict antibiotic consumption in regions where at the one hand detailed information on antibiotic consumption by species is not available, but where at the other hand enough data on how animal populations are structured is given. This is usually the case for all international data sets comparing AMU by country.

Both standardization techniques, direct and indirect, in general represent an artificial process. For the direct standardization livestock demographics of the standard population are assumed to be livestock demographics of the study population, i.e., treatment is compared in similar populations. Vice versa the same applies to the indirect standardization, where the treatment regime is assumed the same, which implies the comparison of a population with an expected result under a given treatment regimen. This indirect approach is more closely related to the intended purposes in the context of a harmonized approach of cross-border AMU comparisons. While interpreting the present results, it should be noted that each antibiotic treatment is composed of different drugs and components, respectively. Because the applied amounts of active ingredients are summed up, these differences in potencies are not considered in the outcome.

Regardless of which of the two methods is used, applying the standardization technique leads to a control for confounding biases resulting from different livestock demographics. After considering the stratification, the previously existing bias has decreased. Accordingly, the calculation indicates that there is an effect, and its extent can only be determined with detailed information at the level of the individual animal species. In order to integrate evaluations of this kind into the reports of ESVAC, appropriate information on the AMU at species level is required in addition to the already existing “estimated PCU in tons of the population by species and country,” which could serve as data basis for the strata. This information could be derived in the form of active substance-related recommended dose per species (Defined Daily Doses Animal) either from country-specific summaries of product characteristics or from scientific studies such as Sjölund et al. (31). As long as more detailed country specific antibiotic consumption data are not available, the proposed standardization technique could serve as an interim solution to improve the comparison of AMU of livestock in different countries.

If structural differences within a population are not considered, there is a risk of possible bias in the comparison of antimicrobial consumption data of individual countries at the general PCU level. Table 7 clearly shows the variability in the proportions of livestock animals within selected animal species and selected member states of the EU (27). The application of the methodology to selected European livestock demographics and AMU-data shows that the standardization technique has substantial effects on the ratios calculated.

Within the example, more than half of Denmark's biomass is comprised of pigs, which have higher AMU than, for example, cattle. In contrast to this the German population is 25% pigs only. As a consequence, the resulting treatment ratio of 2.0 comparing Denmark and Germany is 2-fold higher, which is largely due to the higher proportion of pigs and not to different treatment regimes. This bias could be reduced with standardization. If it is assumed that in Denmark is the same proportion of animals as in Germany, the CTF is 1.2 only. However, it should also be taken into account that regions with a high livestock density may have a higher consumption of antibiotics because of greater health problems caused by high density of farms. For the example, this means that Denmark could have a lower AMU if the density of pig farms would be lower. Consequently, this would also have a reducing effect on the CTF. If on the other hand, in Denmark the same treatment is assumed as in Germany the STR is 1.2. Both standardized rates therefore approach 1, i.e., the non-standardized treatment ratio is strictly biased and heavily overestimate the true ratio of both countries. Taking into account that within the example 85 % of the biomass o France is made up of cattle, the results of the comparison between France and Germany can be interpreted equivalently.

This implies that comparing countries and disregarding the corresponding proportions of the individual animal species may lead to biased results in terms of the overall assessment of antibiotic consumption. By taking into account the livestock demographics or transfer of treatment regimes, a potential confounding can be reduced.

## Conclusions

Within this paper, comparisons of antimicrobial usage in different livestock populations showed that the structural composition of the livestock population has an impact on total consumption. Therefore, we recommend an indirect standardization method for cross-population comparisons to achieve a more compelling comparability and obtain deeper insight in the context of monitoring antibiotic consumption in livestock populations. Corresponding detailed information on antimicrobial usage by species should be made available for this type of stratification so that animal populations can be structured accordingly well.

## Data Availability Statement

The datasets generated for this study are available on request to the corresponding author.

## Ethics Statement

The data used for the VetCAb-example are based on mandatory application and delivery forms, which were provided voluntarily by farmers and veterinarians, signing individual written consent for the data to be used by the study team only. Our research does not involve any regulated animals, and there were no scientific procedures performed on animals of any kind. For this reason, formal approval by an ethical committee was not necessary under the provisions of the German regulations.

## Author Contributions

KH and LK: conceptualization, formal analysis, investigation, and writing—original draft. KH and MH: data curation and validation. LK: funding acquisition and supervision. KH, CV, and LK: methodology. KH and SK: project administration. MH: software. KH, CV, SK, and LK: writing—review and editing. All authors contributed to the article and approved the submitted version.

## Conflict of Interest

The authors declare that the research was conducted in the absence of any commercial or financial relationships that could be construed as a potential conflict of interest.

## Abbreviations

AMU: Antimicrobial usage
CTF: Comparative Treatment Figure
EMA: European Medicines Agency
ESVAC: European Surveillance of Veterinary Antimicrobial Consumption
PCU: Population Correction Unit
STR: Standardized Treatment Ratio
TR: Treatment Ratio
VetCAb-S: Veterinary Consumption of Antibiotics – Sentinel.
